# Phytostabilization of Mine Tailings in Arid and Semiarid Environments—An Emerging Remediation Technology

**DOI:** 10.1289/ehp.10608

**Published:** 2007-12-17

**Authors:** Monica O. Mendez, Raina M. Maier

**Affiliations:** Department of Soil, Water, and Environmental Science, University of Arizona, Tucson, Arizona, USA

**Keywords:** arid, mine tailings, phytostabilization, remediation, revegetation, semiarid

## Abstract

**Objective:**

Unreclaimed mine tailings sites are a worldwide problem, with thousands of unvegetated, exposed tailings piles presenting a source of contamination for nearby communities. Tailings disposal sites in arid and semiarid environments are especially subject to eolian dispersion and water erosion. Phytostabilization, the use of plants for *in situ* stabilization of tailings and metal contaminants, is a feasible alternative to costly remediation practices. In this review we emphasize considerations for phytostabilization of mine tailings in arid and semiarid environments, as well as issues impeding its long-term success.

**Data sources:**

We reviewed literature addressing mine closures and revegetation of mine tailings, along with publications evaluating plant ecology, microbial ecology, and soil properties of mine tailings.

**Data extraction:**

Data were extracted from peer-reviewed articles and books identified in Web of Science and Agricola databases, and publications available through the U.S. Department of Agriculture, U.S. Environmental Protection Agency, and the United Nations Environment Programme.

**Data synthesis:**

Harsh climatic conditions in arid and semiarid environments along with the innate properties of mine tailings require specific considerations. Plants suitable for phytostabilization must be native, be drought-, salt-, and metal-tolerant, and should limit shoot metal accumulation. Factors for evaluating metal accumulation and toxicity issues are presented. Also reviewed are aspects of implementing phytostabilization, including plant growth stage, amendments, irrigation, and evaluation.

**Conclusions:**

Phytostabilization of mine tailings is a promising remedial technology but requires further research to identify factors affecting its long-term success by expanding knowledge of suitable plant species and mine tailings chemistry in ongoing field trials.

Mine tailings disposal sites from either inactive or abandoned mine sites are prevalent in arid and semiarid regions throughout the world. Major areas include northern Mexico and the Western United States, the Pacific coast of South America (Chile and Peru), southwestern Spain, western India, South Africa, and Australia (Munshower 1994; Tordoff et al. 2000). The global impact of such mine tailings disposal sites is enormous, as unreclaimed mining sites generally remain unvegetated for tens to hundreds of years, and exposed tailings can spread over tens of hectares via eolian dispersion and water erosion [Gonzalez and Gonzalez-Chavez 2006; Morris et al. 2003; Munshower 1994; U.S. Environmental Protection Agency (U.S. EPA) 2004; Warhurst 2000].

Mine tailings, or mill tailings, are the materials remaining after extraction and beneficiation of ores. What prevents the natural revegetation of mine tailings? It is generally a combination of factors beginning with metal toxicity. Tailings are characterized by elevated concentrations of metals such as arsenic, cadmium, copper, manganese, lead, and zinc (1–50 g/kg) (Boulet and Larocque 1998; Bradshaw et al. 1978; Walder and Chavez 1995). Further, tailings contain no organic matter or macronutrients, and usually exhibit acidic pH, although some tailings may be alkaline (Johnson and Bradshaw 1977; Krzaklewski and Pietrzykowski 2002). For these reasons, tailings remain without normal soil structure and support a severely stressed heterotrophic microbial community (Mendez et al. 2007; Southam and Beveridge 1992). Hence, the microbial community is extremely low in species richness and carbon utilization diversity compared with uncontaminated soil (Moynahan et al. 2002). Furthermore, autotrophic iron- and sulfur-oxidizing bacteria dominate the microbial community in mine tailings and are associated with plant death in acidic tailings (Schippers et al. 2000).

In arid and semiarid regions, plant establishment on mine tailings is further impeded by a number of physicochemical factors including extreme temperatures especially at the tailings surface, low precipitation, and high winds. These factors contribute to the development of extremely high salt concentrations ranging up to 22 dS/m due to high evaporation and low water infiltration (Munshower 1994).

## Emerging issues

Disposal of mine wastes historically involved either returning the materials to the mining site; dumping into the ocean, a stream, or lake; or placing them into a receiving pond. Today, surface containment of tailings within embankments remains a commonly used approach. In 1995 it was estimated that on an annual basis over 700 million kg of metals in mine tailings were disposed on land (Warhurst 2000). Alternatively, tailings may be returned to the mine (in-pit storage or backfilling) or mixed with coarse mine waste (co-disposal). In arid and semiarid regions, dry-stacking facilities are most common wherein tailings are dried, spread out, and compacted. However, they remain unstable and subject to eolian dispersion and water erosion with the potential to contaminate nearby communities and environmentally sensitive areas (Gonzalez and Gonzalez-Chavez 2006; Schwegler 2006). Some countries mandate mining companies to remediate or contain tailings piles, whereas others have no such requirements and still allow dumping of mine tailings into bodies of water, thus escalating the existing problem of thousands of abandoned mine tailings sites worldwide (Coates 2005; Munshower 1994).

The construction of above-ground impoundments for mine tailings storage is increasingly problematic in arid and semiarid regions. Prevention of wind erosion by surface-wetting is not practical in such environments, especially after closure of mining operations. Therefore, tailings are a significant source of air pollution in the form of particulate matter measured in fractions of ≤ 10 μm (PM_10_) and ≤ 2.5 μm (PM_2.5_) in aerodynamic diameter (Schwegler 2006). Short-term exposure to particulates (PM_2.5/10_) can lead to illness and even premature death in people with heart or lung disease, respiratory conditions, and decreased lung function, whereas long-term exposure to fine particles can accelerate lung cancer and cause chronic respiratory disease in children (U.S. EPA 2006). Measurements of PM_2.5/10_ are monitored in recently established mining operations in some countries but are not monitored for abandoned mine tailings disposal sites despite a link to respiratory health problems and the proximity of disposal sites to human populations.

## Conventional remediation

Conventional technologies for remediation of mine tailings have focused on physical and chemical stabilization. Physical stabilization entails covering mine waste with an innocuous material, generally waste rock from mining operations, gravel, topsoil from an adjacent site, or a clay capping, to reduce wind and water erosion. These solutions are often temporary in nature because of the impermanence of the capping process (Johnson and Bradshaw 1977). For example, clay caps in arid and semiarid environments crack from the wetting–drying cycles and poor consolidation of the tailings due to their high salinity (Newson and Fahey 2003; Swanson et al. 1997). Chemical stabilization aims to prevent wind and water erosion using a chemical agent such as a lignin sulfonate or a resinous adhesive to form a crust over the tailings, also a temporary stabilization technique, as these crusts can eventually fail (Tordoff et al. 2000). Recently, reprocessing historic tailings materials using more advanced technologies to reduce metal concentrations and toxicity has been considered and is economical in some cases (Warhurst 2000). However, the tailings material is still left after reprocessing and must be stabilized in some way. In general, traditional remediation techniques range from approximately US$1.50–450 per m^3^ for mine tailings contained by waste rock or cemented backfilling (Berti and Cunningham 2000; Evans and Willgoose 2000). An emerging remediation technology, phytostabilization, can reduce this cost to an estimated US$0.40–26 per m^3^ for revegetation alone or for lined and revegetated repositories (Ford and Walker 2003).

## Phytostabilization as a remediation strategy

**Figure 1:**
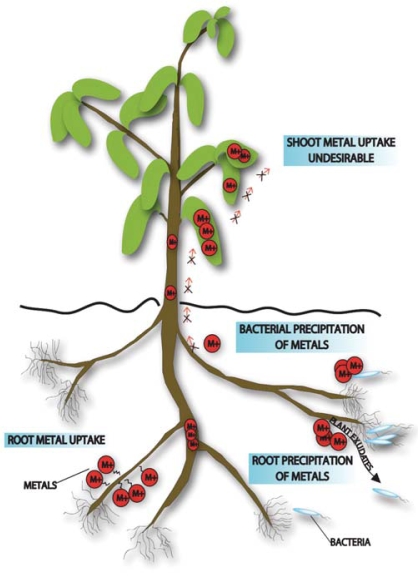
Schematic showing phytostabilization mechanisms including precipitation of metals by bacterial and root surfaces, precipitation of metals by bacterial and root exudates, bacterial uptake and sequestration of metals, and root uptake of metals. In phytostabilization, accumulation of metals in plant shoot tissues is undesirable.

Phytostabilization creates a vegetative cap for the long-term stabilization and containment of the tailings. The plant canopy serves to reduce eolian dispersion whereas plant roots prevent water erosion, immobilize metals by adsorption or accumulation, and provide a rhizosphere wherein metals precipitate and stabilize. Unlike phytoextraction, or hyperaccumulation of metals into shoot/root tissues (Ernst 2005), phytostabilization primarily focuses on sequestration of the metals within the rhizosphere but not in plant tissues (Figure 1). Consequently, metals become less bioavailable and livestock, wildlife, and human exposure is reduced (Cunningham et al. 1995; Munshower 1994; Wong 2003).

Although phytostabilization of mine tailings sites in arid and semiarid regions has been experimented with by mining companies, documentation of this remediation technology only occasionally appears in published literature, so general understanding of this technology is limited. In this review we address the current knowledge of phytostabilization in arid and semiarid environments as well as potential problems that impact the long-term success of this technology.

## Phytostabilization of Mine Tailings in Arid and Semiarid Environments

Phytostabilization of mine tailings in arid and semiarid environments involves the use of drought-, salt-, and metal-tolerant plants for immobilization of heavy metals in the tailings substrate. In theory, metal bioavailability (and hence toxicity) will decrease as plants facilitate the precipitation of metals to less soluble forms, for example, metal sulfides or metal carbonates, complex metals with organic products, sorb metals onto root surfaces, and accumulate metals into root tissues (Cunningham et al. 1995; Wong 2003). Furthermore, the presence of plants in mine tailings enhances the heterotrophic microbial community, which may, in turn, promote plant growth and participate in metal stabilization (Glick 2003; Mendez et al. 2007; Mummey et al. 2002). The ultimate objective for successful phytostabilization is the long-term succession of the plant community in mine tailings to promote soil development processes, microbial diversity, and finally, to restore soil ecosystem functions to a state of self-sustainability.

### Plant candidate requirements for phytostabilization

Phytostabilization of mine tailings in arid and semiarid environments requires establishing a diverse plant community by including drought-, metal-, and salt-tolerant plants that do not hyperaccumulate metals of concern into shoot tissues. Candidates for phytostabilization ideally should be native to the area in which the mine tailings are found, as they have evolved survival mechanisms appropriate to the harsh climate of arid and semiarid environments. A secondary but also important consideration is that the use of native plants avoids introduction of nonnative and potentially invasive species that may result in decreasing regional plant diversity. To date, many field trials have not taken advantage of native plant diversity, resulting in poor plant colonization and soil conditions.

Selection of a variety of perennial grasses, shrubs, and trees for revegetation of mine tailings is important for phytostabilization. Grasses provide a quick ground cover and temporarily limit eolian dispersion of tailings, whereas shrubs and trees become established (Williams and Currey 2002). Shrubs and trees provide an extensive canopy cover and establish a deeper root network to prevent erosion over the long term. Furthermore, inclusion of the various growth habits maintains species and functional diversity. Shrubs or trees provide a high nutrient environment for grasses while reducing moisture stress and improving soil physical characteristics in arid and semiarid climates (Belsky et al. 1989; Tiedemann and Klemmedson 1973, 2004). Additionally, the establishment of different functional groups increases plant productivity and yield. Although a few plants may eventually dominate the ecosystem because of selective pressures, the presence and effect of less abundant species is still significant in promoting a self-sustainable ecosystem (Tilman et al. 2001).

Arid and semiarid soils are often saline, as evaporation rates exceed rainfall and natural salts originate from saline rainfall, unweathered minerals, and fossil salts. In mine tailings operations within arid and semiarid environments, saline groundwater is often used in the beneficiation process, which becomes hypersaline as it is recycled throughout ore processing. As tailings dry, salt crusts form on the surface (Munshower 1994; Newson and Fahey 2003). Therefore, halophytes (salt-tolerant plants) are especially valuable in phytostabilization. Members of the Chenopodiaceae family, specifically *Atriplex* spp., are highly salt tolerant and serve as pioneer species on mine tailings in semiarid western Australia and are used in revegetation of mine tailings in the Western United States (Glenn et al. 2001; Jefferson 2004; Rosario et al. 2007). Other halophytic shrubs recommended in the Western United States are creosote bush (*Larrea tridentate* DC., Zygophyllaceae) and desert broom (*Baccharis sarothroides* Gray, Asteraceae). Also, leguminous trees that serve as a nitrogen supply such as *Acacia* spp. and *Prosopis* spp. have been reported as successfully colonizing mine tailings in the Western United States (Day et al. 1980).

Plants used for phytostabilization must be metallophytes (metal-tolerant plants) but ones that do not accumulate or limit metal accumulation to root tissues. Although metallophytes have developed mechanisms to impede translocation of metals in the above-ground plant mass, there may still be an excessively high concentration of metals in the shoot material. There are several ways to measure and express metal accumulation in plants. These include *a*) bioconcentration factor (BF) or accumulation factor (AF) = (total element concentration in shoot tissue ÷ total element concentration in mine tailings) and *b*) translocation factor (TF) or shoot:root (S:R) ratio = (total element concentration in shoot tissue ÷ total element concentration in the root tissue).

Ideally these values would be << 1, but they should not exceed a ratio of 1, which would indicate that the plant is useful for phytoextraction (accumulation of metals in shoot tissue) but should not be used in phytostabilization (Brooks 1998). Surveys of native plants colonizing mine tailings have provided promising information, especially plant families of colonizers with relatively low metal accumulation in above-ground tissues (Table 1).

In addition to the above-mentioned metal accumulation ratios, several metal concentration guidelines can be used to help evaluate metal toxicity issues that may arise during phytostabilization (Table 2). The first is soil plant toxicity levels, which can provide a guide in evaluating metal tolerance (Kataba-Pendias and Pendias 2001; Mulvey and Elliott 2000; Munshower 1994). The second is plant leaf tissue toxicity limits, which can help assess the long-term potential for plant establishment. Last, domestic animal toxicity limits can be compared with above-ground metal accumulation, as foragers, including cattle and other wildlife, may consume these plants [National Research Council (NRC) 2005; Wood et al. 1995]. Unfortunately, metal accumulation in field trials has not been thoroughly documented (Johansson et al. 2005). Thus, identification of suitable phytostabilization plant candidates and understanding their metal accumulation patterns are areas in which additional research is critically needed for practitioners in the field.

## Implementation of Phytostabilization

### Seeds versus transplants

In general, direct seeding produces a more patchy vegetation cover than using transplants. However, although the use of transplants produces better results, this approach is more labor intensive, and this is one of the factors that must be taken into consideration for each site. Further, seeding can be successful as demonstrated by a tin mine tailings site in Zimbabwe in which 40% vegetation cover was achieved with a mixture of grasses, herbaceous legumes, and trees and a single treatment of NPK fertilizer (Piha et al. 1995).

One recent study examined metal uptake by *Lygeum spartum* grown from seeds or rhizomes in tailings in a greenhouse study (Conesa et al. 2007). The plants grown from seeds took up significantly more metal than plants grown from rhizomes. However, plants collected from the tailings site itself showed one order of magnitude less metal accumulation. The authors point out that greenhouse conditions (higher moisture content and homogeneity of the tailings) likely influenced the results of the study.

### Amendments

Because the addition of topsoil amended with organic matter and nutrients is not economical for extensive mine tailings sites, organic amendments are generally used as a substitute. Organic amendments help to immediately decrease metal bioavailability, provide a slow-release fertilizer, and serve as a microbial inoculum. In addition, organic matter improves soil structure, reduces erosion, and increases infiltration. The organic matter may be composed of wood chips, straw, biosolids, composted municipal waste, or manure (Munshower 1994). The carbon-to-nitrogen (C:N) ratio of the organic amendment should range from 12:1 to 20:1 to prevent high rates of organic matter decomposition and nitrogen consumption by the microbial community. For example, uncomposted organic amendments such as wood-chips contain a high C:N ratio; therefore, nitrogen may become immobilized and impede long-term plant establishment (Van Rensburg and Morgenthal 2004). The addition of commercial compost to mine tailings has been shown to enhance plant growth in greenhouse trials (Mendez et al. 2007; Munshower 1994; Schippers et al. 2000; Schroeder et al. 2005; Southam and Beveridge 1992). As well as pH levels increasing with compost amendment, there is a decrease in the number of iron- and sulfur-oxidizers attributed to acid production and vegetation death in pyritic tailings. Furthermore, compost can increase the water-holding capacity and cation exchange capacity of mine tailings.

Biosolids have also been used as an amendment to ameliorate the harsh conditions of mine tailings. For example, biosolids successfully increased plant growth in a gold mine tailings field trial in New Zealand (Mains et al. 2006a). Anaerobically digested biosolids are preferred to aerobically treated biosolids because of higher nitrogen content and a greater enhancement of plant growth as tested in a copper mine tailings site (McNearny 1998). However, biosolids may contain phytotoxic levels of metals, depending on the source of the material (Munshower 1994). Also, biosolids amendment is not generally desirable in ecologically sensitive areas.

One area that requires additional research is the potential for the acceleration of metal leaching through the addition of compost or biosolids (Burckhard et al. 1995; Pond et al. 2005). Laboratory studies have shown the potential for enhanced leaching of Zn from mine tailings after addition of model organic acids, for example, oxalic or citric acid, that are found in the rhizosphere (Burckhard et al. 1995). A second study examined leaching of Cu, nickel, and Zn from biosolid-amended tailings from four sites in southern Arizona. This study was performed under laboratory conditions, and results showed that for three of the tailings, which were circumneutral in pH, metal leaching increased only slightly. For the fourth, acidic tailing, metal leaching increased up to 3-fold for Cu and up to 100-fold for Zn. It should be noted that both these studies were performed over the short term and in the absence of plants.

Addition of inorganic fertilizers should be limited, as native vegetation used for phytostabilization of mine tailings in arid and semiarid environments tends to be adapted to low nutrients and tends to respond differently to fertilizer inputs (Piha et al. 1995). Furthermore, if organic amendments are added, there is likely a sufficient or near sufficient concentration of nutrients already present (Munshower 1994; Van Rensburg and Morgenthal 2004). One exception to this is phosphorous fertilizers, which may be necessary to alleviate phosphorous deficiency due to the formation of insoluble metal-phosphates; thus it is important to consider phosphorous fertilizers to alleviate phosphate deficiency. However, the addition of phosphorous amendments can increase arsenic uptake into plants as well as leaching in mine tailings because phosphate behaves chemically similar to arsenate (Mains et al. 2006b). In extremely acidic mine tailings, lime may be required to neutralize acidification; however, without organic matter addition, the site may require continuous inputs of lime to maintain a pH > 5 (Munshower 1994).

### Irrigation

Although drought-tolerant plants must be used in phytostabilization, initial irrigation is usually required to aid plant establishment. If seeds are directly sown into mine tailings, irrigation is especially crucial. However, with exceptional planning, remote locations may be seeded by taking advantage of seasonal rain (Piha et al. 1995). Drip irrigation for at least 3–6 months or until plants become established has proven to be successful in revegetation of mine tailings (Tordoff et al. 2000; Williams and Currey 2002). However, irrigation should be limited for both cost and dependence of the plant community on the availability of water (Munshower 1994).

### Evaluation of successful revegetation

The majority of phytostabilization studies in arid and semiarid environments have focused on plant growth variables such as plant biomass and percent cover; however, other methods of evaluating successful revegetation must be taken into consideration for long-term rehabilitation of mine tailings (Appendix 1). For example, plants should be able to self-propagate successfully with no additional inputs. Also, plant species that were not seeded or transplanted should begin to colonize the area. As previously mentioned, the above-ground plant biomass may be a source of metal exposure for foraging animals (Castro-Larragoitia et al. 1997; Wood et al. 1995). Therefore, domestic animal toxicity limits must be observed to prevent further contamination of the ecosystem. In addition, microbial communities have been largely ignored in field studies within arid and semiarid environments (Mummey et al. 2002). Yet, the heterotrophic microbial community can be linked to plant establishment, and plant–microbe interactions are important for promoting nutrient cycling, soil aggregation, and plant nutrient uptake (Bearden and Petersen 2000; Mendez et al. 2007; Moynahan et al. 2002; Rosario et al. 2007). Heterotrophic microbial numbers must increase, replacing the dominant presence of autotrophic iron- and sulfur-oxidizing bacteria, a community of microorganisms that plays a critical role in acidification of the tailings. Further, soil structure should improve with a recovery of the heterotrophic microorganisms and recycling of organic matter.

One critical aspect largely missing from the published literature is information about the long-term success of phytostabilization. Most studies are terminated after 1–2 years. Although organic amendments are favored for their ability to immediately decrease metal bioavailability, weathering and decomposition of organic residues may ultimately enhance metal mobility (Pierzynski et al. 2002; Pond et al. 2005; Tordoff et al. 2000). For example, Pond et al. (2005) examined both circumneutral and acidic copper mine tailings from Arizona in a simulated weathering study. Addition of biosolids decreased concentrations of copper, nitrate, and sulfate in leachate from the acidic tailings sample but slightly increased copper and arsenic in leachate from the circumneutral sample compared with concentrations in unamended tailings. Further, in short-term studies, metal availability based on plant metal accumulation may be deceiving. For example, a study of a 20-year rehabilitated uranium site in Australia indicated an increase in metal mobility in the soil surface and an increase in plant metal accumulation (Lottermoser et al. 2005). As these studies suggest, additional long-term studies on the chemical state of tailings and plants are essential for determining phytostabilization success.

## Conclusions

Existing phytostabilization studies in arid and semiarid environments are limited and have not addressed several important issues. For example, plant metal accumulation has not been documented in the majority of field studies (Johansson et al. 2005). This is especially important in determining the long-term fate of plant establishment as well as the metal contaminants. In addition, easily accessible information is needed on ranges of metal tolerance in metallophyte species suitable for growth in mine tailings; minimum amounts of organic amendments required for successful plant establishment; and minimum water requirements necessary for successful plant establishment. It is likely that a small pilot study, performed to optimize choice of candidate species and amounts of growth amendments for each specific tailings site, may be necessary to ultimately reduce costs and make implementation of phytostabilization successful.

Finally, the long-term fate of metals in revegetated tailings has not been explored thoroughly. Such information is needed to help evaluate the efficacy of phytostabilization in permanently reducing metal toxicity, in promoting plant succession, and in promoting the formation of soil structure and properties in tailings materials.

In summary, phytostabilization of mine tailings in arid and semiarid regions has promising potential. Studies have indicated that plant establishment on mine tailings is possible, and when successful, helps to reduce erosion processes and enhance soil formation properties. Although it may not be possible to create an ecosystem equivalent to the surrounding uncontaminated area, successful phytostabilization can create a self-sustaining biological cap with an attendant ecosystem that more closely resembles a healthy soil-plant environment. Defining and understanding this process will require long-term studies that explore interactions between plants and the microbial and physical–chemical characteristics of the tailings as they undergo the revegetation process.

## Figures and Tables

**Table 1:** Plant families of potential phytostabilization candidates.

Plant^a^	Metal contaminants	Location	Comment and reference
Anacardiaceae			
*Pistacia terebinthus* Bieberstein	Cu	Cyprus	Field study using chicken fertilizer and 1:1 soil and mine waste (Johansson et al. 2005)
*Schinus molle L.*	Cd, Cu, Mn, Pb, Zn	Mexico	Plant survey (Gonzalez and Gonzalez-Chavez 2006)
Asteraceae			
*Baccharis neglecta* Britt.	As	Mexico	Plant survey (Flores-Tavizon et al. 2003)
*Bidens humilis* H.B.K.	Ag, As, Cd, Cu, Pb, Zn	Ecuador	Plant survey (Bech et al. 2002)
*Isocoma veneta* (Kunth) Greene	Cd, Cu, Mn, Pb, Zn	Mexico	Plant survey (Gonzalez and Gonzalez-Chavez 2006)
*Viguiera linearis* (Cav.) Sch.
Chenopodiaceae			
*Teloxys graveolens* (Willd.) W.A. Weber	Cd, Cu, Mn, Pb, Zn	Mexico	Plant survey (Gonzalez and Gonzalez-Chavez 2006)
*Atriplex lentiformis* (Torr.) S. Wats.	As, Cu, Mn, Pb, Zn	U.S.	Greenhouse study using compost (Mendez et al. 2007)
*Atriplex canescens* (Pursh) Nutt.	As, Hg, Mn, Pb	U.S.	Field study (Rosario et al. 2007)
Euphorbiaceae			
*Euphorbia sp.*	Cd, Cu, Mn, Pb, Zn	Mexico	Plant survey (Gonzalez and Gonzalez-Chavez 2006)
Fabaceae			
*Dalea bicolor* Humb. & Bonpl. ex Willd.	Cd, Cu, Mn, Pb, Zn	Mexico	Plant survey (Gonzalez and Gonzalez-Chavez 2006)
Plumbaginaceae			
*Lygeum spartum* L.	Cu, Pb, Zn	Spain	Plant survey (Conesa et al. 2006)
Poaceae			
*Piptatherum miliaceum* (L.) Coss.	Cu, Pb, Zn	Spain	Plant survey (Conesa et al. 2006)

a Plants listed are native species documented in the respective paper with plant metal accumulation in above-ground biomass that does not exceed domestic animal toxicity limits (NRC 2005).

**Table 2:** Metal toxicity limits (mg/kg).

Toxicity index	As	Cd	Cu	Mn	Ni	Pb	Zn
Soil plant toxicity levels^a^	15	3	200	3,000	90	100–500	400
Plant leaf tissue toxicity limits^b^	5–20	5–30	2–20	400–1,000	10–100	30–100	100–400
Domestic animal toxicity limits^c^	30	10	40	2,000	100	100	500

a Based on total metal concentrations generally toxic to plant growth (Kataba-Pendias and Pendias 2001; Khalid and Tinsley 1980; Mulvey and Elliott 2000; Munshower 1994).

b Based on mean values of toxic levels of metals accumulated in agricultural crops (Kataba-Pendias and Pendias 2001).

c Based on maximum tolerable levels for cattle (NRC 2005).

## Appendix 1. Criteria for evaluating successful revegetation of mine tailings

### Plant criteria

- Biomass and percent cover comparable or exceeding growth in uncontaminated soil
- Self-propagation of introduced plants
- Establishment of native colonizers
- Shoot metal concentrations not exceeding the domestic animal toxicity limits
- Plant survival and productivity maintained for > 10–20 years

### Microbial criteria

- Heterotrophic bacterial and fungal counts increased
- Autotrophic iron- and sulfur-oxidizing bacterial counts decreased

### Soil criteria

- Soil aggregation improved
- Erosion and runoff reduced
- Metal bioavailability and mobility decreased
